# Phospholipase C-β1 and β4 Contribute to Non-Genetic Cell-to-Cell Variability in Histamine-Induced Calcium Signals in HeLa Cells

**DOI:** 10.1371/journal.pone.0086410

**Published:** 2014-01-27

**Authors:** Sachiko Ishida, Toru Matsu-ura, Kiyoko Fukami, Takayuki Michikawa, Katsuhiko Mikoshiba

**Affiliations:** 1 Laboratory for Developmental Neurobiology, RIKEN Brain Science Institute, Wako, Japan; 2 Laboratory of Genome and Biosignal, Tokyo University of Pharmacy and Life Sciences, Tokyo, Japan; 3 Calcium Oscillation Project, ICORP-SORST, Japan Science and Technology Agency, Kawaguchi, Japan; 4 Laboratory for Behavioral Genetics, RIKEN Brain Science Institute, Wako, Japan; University of Debrecen, Hungary

## Abstract

A uniform extracellular stimulus triggers cell-specific patterns of Ca^2+^ signals, even in genetically identical cell populations. However, the underlying mechanism that generates the cell-to-cell variability remains unknown. We monitored cytosolic inositol 1,4,5-trisphosphate (IP_3_) concentration changes using a fluorescent IP_3_ sensor in single HeLa cells showing different patterns of histamine-induced Ca^2+^ oscillations in terms of the time constant of Ca^2+^ spike amplitude decay and the Ca^2+^ oscillation frequency. HeLa cells stimulated with histamine exhibited a considerable variation in the temporal pattern of Ca^2+^ signals and we found that there were cell-specific IP_3_ dynamics depending on the patterns of Ca^2+^ signals. RT-PCR and western blot analyses showed that phospholipase C (PLC)-β1, -β3, -β4, -γ1, -δ3 and -ε were expressed at relatively high levels in HeLa cells. Small interfering RNA-mediated silencing of PLC isozymes revealed that PLC-β1 and PLC-β4 were specifically involved in the histamine-induced IP_3_ increases in HeLa cells. Modulation of IP_3_ dynamics by knockdown or overexpression of the isozymes PLC-β1 and PLC-β4 resulted in specific changes in the characteristics of Ca^2+^ oscillations, such as the time constant of the temporal changes in the Ca^2+^ spike amplitude and the Ca^2+^ oscillation frequency, within the range of the cell-to-cell variability found in wild-type cell populations. These findings indicate that the heterogeneity in the process of IP_3_ production, rather than IP_3_-induced Ca^2+^ release, can cause cell-to-cell variability in the patterns of Ca^2+^ signals and that PLC-β1 and PLC-β4 contribute to generate cell-specific Ca^2+^ signals evoked by G protein-coupled receptor stimulation.

## Introduction

Many extracellular stimuli trigger increases in the cytosolic concentration of Ca^2+^ ([Ca^2+^]) that control a wide range of physiological processes, including fertilization, proliferation, development, learning and memory, contraction, and secretion [1], [2]. In a multitude of cell types, released Ca^2+^ causes oscillatory changes in [Ca^2+^], and the frequency of Ca^2+^ oscillations is correlated with the stimulus intensity [3]–[5], while the time course of an individual Ca^2+^ spike depends on the type of receptor stimulated [6], [7]. The versatility of Ca^2+^ signals is considered to be dependent on the spatiotemporal patterns of intracellular Ca^2+^ signals [8].

Extracellular stimuli, such as growth factors, hormones, and neurotransmitters, activate an intracellular effector molecule, phosphoinositide-specific PLC, that catalyzes the conversion of phosphatidylinositol 4,5-bisphosphate (PIP_2_) to inositol 1,4,5-trisphosphate (IP_3_) and diacylglycerol [9]. IP_3_ acts as a second messenger in many cell types, where its primary effect is to trigger the release of Ca^2+^ ions from intracellular stores and generate cytosolic Ca^2+^ signals. The forms of the Ca^2+^ signals vary from cell to cell, even in clonal populations of genetically identical cells, while individual cells show characteristic and reproducible Ca^2+^ responses in terms of amplitude, frequency, and shape of the transient as well as the latency of onset of the initial Ca^2+^ rise [5]. Therefore, Ca^2+^ signals have been referred to as cell “fingerprints”.

Real-time monitoring of cytosolic IP_3_ in living cells is pivotal for understanding the mechanism that underlies the generation of cell-specific patterns of Ca^2+^ signals. In a previous study, we monitored the cytosolic IP_3_ concentration ([IP_3_]) changes during agonist-evoked Ca^2+^ oscillations in HeLa cells using a genetically-encoded fluorescent IP_3_ sensor IP_3_R-based IP_3_ sensor 1 (IRIS-1) [10]. The observed IP_3_ dynamics were unexpectedly complex because [IP_3_] did not return to its basal level during the intervals between Ca^2+^ spikes, and IP_3_ gradually accumulated in the cytosol with little or no fluctuation during cytosolic Ca^2+^ oscillations [10]. Manipulation of the cytosolic Ca^2+^ increases during agonist application indicated the presence of both Ca^2+^-dependent and Ca^2+^-independent components for IP_3_ generation in this cell type [10]. The molecular natures of these components and, more fundamentally, the roles of IP_3_ dynamics in the generation of the cell-specific patterns of Ca^2+^ signals remain elusive.

There are six families of mammalian PLC enzymes (PLC-β, -γ, -δ, -ε, -ζ, and -η) consisting of 13 isoforms in humans [11], [12]. PLC-β is activated by the Gα and Gβγ subunits of heterotrimeric G proteins downstream of G protein-coupled receptors (GPCRs). PLC-γ is activated through receptor and non-receptor tyrosine kinases. PLC-δ is activated by cytosolic Ca^2+^ and the high-molecular-weight G protein Gh. PLC-ε is regulated directly by small GTPases from the Ras family, and its enzymatic activity can be stimulated by subunits of heterotrimeric G proteins and small GTPases from the Rho family. PLC-ζ was identified as a sperm-specific PLC, and shows extremely high Ca^2+^ sensitivity for its enzymatic activity compared with the other PLC isoforms. PLC-η is suggested to be activated through GPCR stimulation. Although various tissues express multiple PLC isozymes, the effects of the individual PLC isozymes on the generation of Ca^2+^ signals are poorly understood.

In this study, we measured the IP_3_ dynamics using IRIS-1 in cells showing different patterns of Ca^2+^ signals within a clonal population of genetically identical HeLa cells stimulated with histamine. The IP_3_ dynamics were characteristically different depending on the patterns of cellular Ca^2+^ signals, suggesting that the heterogeneity in IP_3_ production contributes to the cell-to-cell variability observed in Ca^2+^ signals. We combined an RNAi technique with the real-time IP_3_ imaging technique to identify the PLC isozymes involved in the generation of IP_3_ production in this cell type, and found that at least two isozymes in the PLC-β family, PLC-β1 and PLC-β4, are involved in histamine-induced IP_3_ increases in HeLa cells. RNAi-mediated silencing and overexpression experiments for PLC-β1 and PLC-β4 revealed that these isozymes differentially contribute to the determination of the characteristic parameters of Ca^2+^ signals, i.e. the Ca^2+^ oscillation frequency and the time constant of Ca^2+^ spike amplitude decay. These findings provide important clues toward understanding the molecular mechanism that underlies the generation of cell-specific patterns of Ca^2+^ signals in genetically identical cell populations.

## Results

### Reproducible Cell-specific Patterns of [Ca^2+^] and [IP_3_] Changes in HeLa Cells Stimulated with 3 µM Histamine

**Figure 1:**
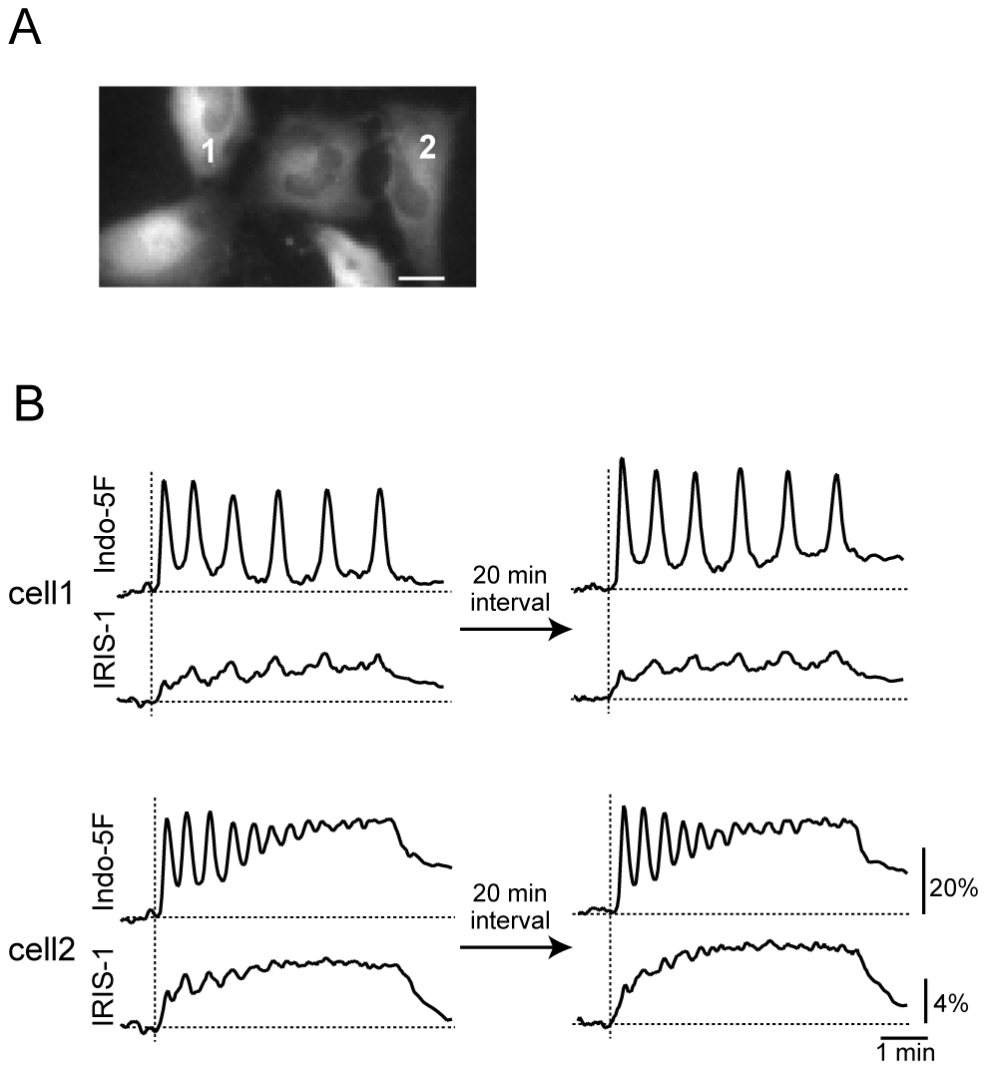
Reproducible cell-specific patterns in Ca^2+^ and IP_3_ responses in HeLa cells stimulated with histamine. (A) Venus fluorescence image of IRIS-1-expressing HeLa cells before histamine stimulation. Bar, 20 µm. (B) Changes in 460–510 nm emission of Indo-5F signals (F/F_0,_ where F_0_ is the basal level of F; top) and ECFP/Venus emission ratio of IRIS-1 signals (ΔR/R_0_; ΔR was defined as R – R_0_, where R_0_ is the basal level of R; bottom) after repeated additions of 3 µM histamine with a 20-min interval in two different cells within the same field of view shown in (A). The horizontal broken lines indicate the baseline levels of the IRIS-1 and Indo-5F signals. The vertical broken lines indicate the onsets of stimulation.

To examine whether the cell-specific patterns of Ca^2+^ oscillations were accompanied by characteristic IP_3_ dynamics in individual cells, we performed simultaneous monitoring of [Ca^2+^] and [IP_3_] at the single-cell level. Indo-5F was loaded into IRIS-1-expressing HeLa cells and the cells were stimulated twice with 3 µM histamine with an interval of approximately 20 min. Consistent with previous studies [5], [13]–[15], there was marked intercellular heterogeneity in the Ca^2+^ dynamics, which varied in the pattern (sustained oscillations or damped oscillations) and the frequency of Ca^2+^ oscillations (for more details, see below), even within the same microscopic field of view (Fig. 1A). These cell-specific Ca^2+^ patterns were reproducible upon repetitive histamine additions (Fig. 1B), as observed in different cell types [5], [13]–[15], suggesting that individual HeLa cells have characteristic Ca^2+^ dynamics. As shown in Fig. 1B, the patterns of IP_3_ dynamics also differed between cells. In cells showing sustained Ca^2+^ oscillations (*e.g*. cell 1 in Fig. 1B), IP_3_ was maintained at relatively low levels during the period of stimulation, and fluctuations in [IP_3_] that synchronized with Ca^2+^ spikes were observed with almost constant amplitudes. In cells showing damped Ca^2+^ oscillations (*e.g*. cell 2 in Fig. 1B), the initial increase in [IP_3_] was relatively high and the IP_3_ level gradually increased during the period of stimulation. [IP_3_] fluctuations that synchronized with Ca^2+^ spikes were also observed in cells showing damped Ca^2+^ oscillations, but the amplitude of these [IP_3_] fluctuations gradually decreased depending on the amplitude of the Ca^2+^ spikes. In both cell types, the IP_3_ dynamics were reproducible (Fig. 1B). These findings indicate that the IP_3_ dynamics are characteristic of individual cells, and that the IP_3_ dynamics may be involved in the determination of cell-specific Ca^2+^ responses.

### [Ca^2+^] and [IP_3_] Dynamics Observed in Cells Stimulated with Different Concentrations of Histamine

**Figure 2:**
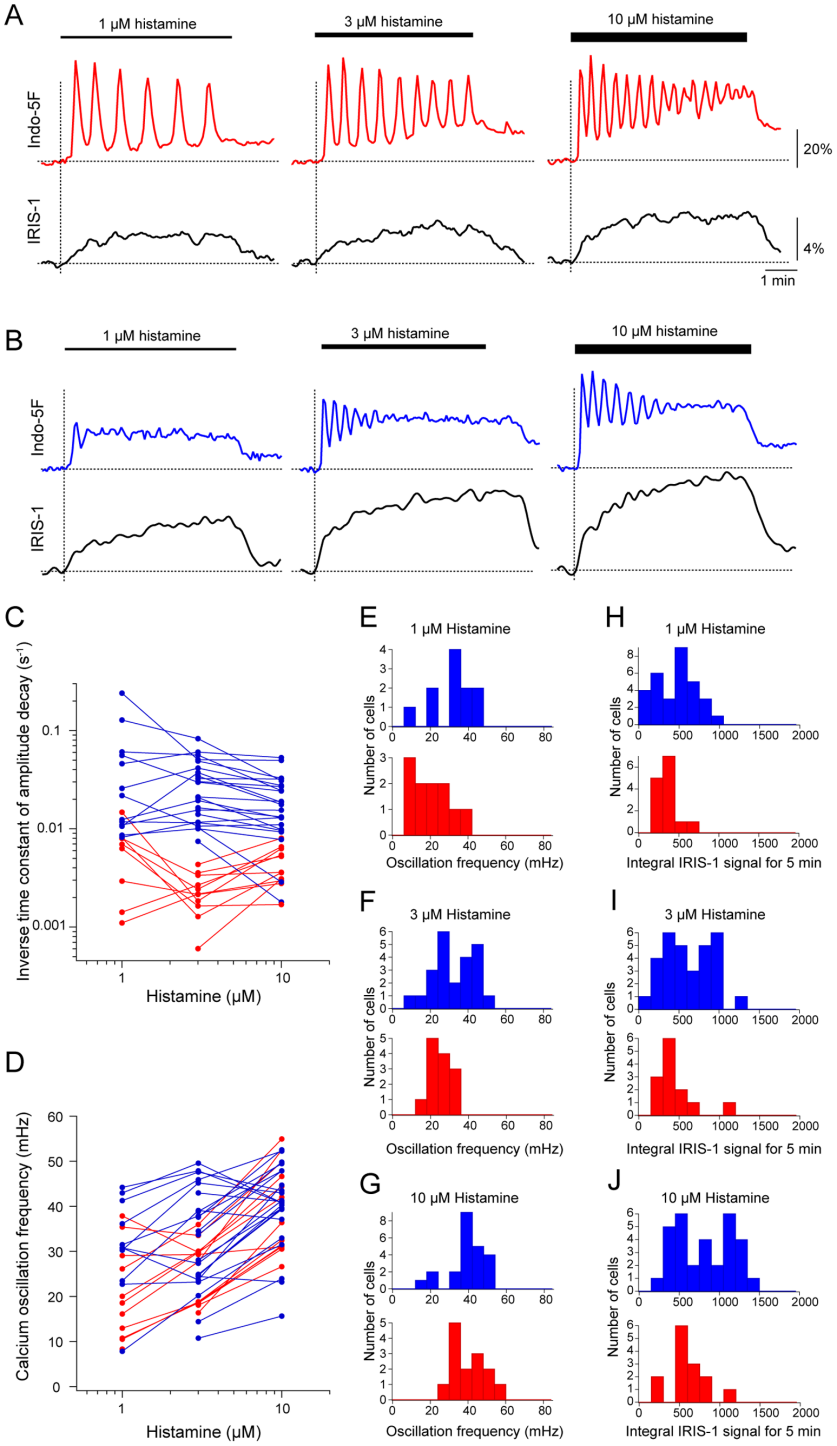
Classification of cells depending on the time constants of peak amplitude decay of Ca^2+^ spikes. Representative traces of Indo-5F signal changes (F/F_0_; top) and IRIS-1 signal changes (ΔR/R_0_; bottom) in cells showing sustained Ca^2+^ oscillations (S-cell) (A) and damped oscillations (D-cell) (B). Three different concentrations of histamine (1, 3, and 10 µM) were sequentially applied to the same cells with an interval of 20 min. (C) Relationships between the histamine concentrations and the inverse time constants of Ca^2+^ amplitude decay in S-cells (red) and D-cells (blue). (D) Relationships between the histamine concentrations and the Ca^2+^ oscillation frequencies observed in S-cells (red) and D-cells (blue). (E–G) Comparisons of the Ca^2+^ oscillation frequencies between S-cells (red) and D-cells (blue). The histamine concentrations were 1 µM (E), 3 µM (F), and 10 µM (G). (H–J) Comparisons of integrated IRIS-1 signals between S-cells (red) and D-cells (blue). The histamine concentrations were 1 µM (H), 3 µM (I), and 10 µM (J).

As shown in Fig. 1, there were two prominent types of cells depending on their temporal patterns of [Ca^2+^] changes after stimulation with 3 µM histamine. To investigate whether these differences arose from heterogeneity in the histamine sensitivity of the cells, as proposed by Rooney et al. [15], or from other components in the signal transduction pathway, we measured the [Ca^2+^] and [IP_3_] changes in the same cells stimulated with three different concentrations of histamine. Representative results are shown in Fig. 2A (cells showing sustained Ca^2+^ oscillations at 3 µM histamine) and Fig. 2B (cells showing damped Ca^2+^ oscillations at 3 µM histamine). To analyze the results quantitatively, we measured the time constants for the progressive decrease in the amplitude of Ca^2+^ spikes (Fig. S1A). Sustained oscillations had larger time constants of Ca^2+^ spike amplitude decay, while damped oscillations had smaller time constants of Ca^2+^ spike amplitude decay. Fig. 2C shows the relationships between the time constants and the histamine concentrations applied. There were large cell-to-cell varieties for all three histamine concentrations examined, and the inverse time constants varied from less than 0.001 s^−1^ (stable sustained oscillations) to 0.3 s^−1^ (rapidly damped oscillations). There was no obvious dependence of the time constant on the histamine concentration, and individual cells showed similar time constants irrespective of the histamine concentration (Fig. 2C). These findings suggest that a difference in histamine sensitivity is not the primary mechanism for the generation of different patterns of Ca^2+^ signals and that the time constants are characteristic of individual cells. Because there were two peaks in the histogram of the time constants observed in cells stimulated with 3 µM histamine (Fig. S1B), we classified the cells into two groups, namely S-cells (cells showing sustained oscillations: red in Fig. 2C) and D-cells (cells showing damped oscillations: blue in Fig. 2C), depending on the time constants of Ca^2+^ amplitude decay observed at 3 µM histamine with a threshold of 0.0056 s^−1^.

Fig. 2D shows the relationships between the histamine concentrations and the Ca^2+^ oscillation frequencies in S-cells (red) and D-cells (blue). The frequency increased depending on the concentration of histamine, with slopes of 2.2±1.1 mHz/µM histamine (mean ± SD, *n* = 14) for S-cells and 1.2±1.0 mHz/µM histamine (mean ± SD, *n* = 23) for D-cells. There were large cell-to-cell variabilities in the Ca^2+^ oscillation frequencies, but the frequencies in S-cells tended to be smaller than those in D-cells (Fig. 2D–G). We detected significant differences (P<0.05, Student's *t*-test) in the Ca^2+^ oscillation frequencies between S-cells and D-cells following stimulation with 1 µM histamine (mean ± SD: 20.5±10.3 mHz, *n* = 11, for S-cells vs. 31.1±10.6 mHz, *n* = 11, for D-cells) and 3 µM histamine (mean ± SD: 25.0±6.9 mHz, *n* = 13, for S-cells vs. 32.5±11.2 mHz, *n* = 23, for D-cells) (Fig. 2E and F). These findings indicate the presence of a correlation between the interval of Ca^2+^ spikes and the time dependence of the change in the amplitude of Ca^2+^ spikes.

Representative traces showed that the dependence of the Ca^2+^ spike amplitude on the histamine concentration differed between S-cells and D-cells (Fig. 2A and B). We quantified the peak amplitudes of the first Ca^2+^ spike following stimulation with three different concentrations of histamine (Fig. S2A and B). The amplitude of the first spike of S-cells remained almost constant irrespective of the histamine concentration applied, while that of D-cells was dependent on the histamine concentration (Fig. S2B). These results suggest that the histamine concentration-dependence of the amplitude of the first Ca^2+^ spikes is tightly coupled to the rate of changes in the amplitude of the following Ca^2+^ spikes in HeLa cells.

Fig. 2H–J shows histograms of the integrals of the IP_3_ signals during stimulation with the three different concentrations of histamine. As shown in Fig. S2C, the mean values of the integrated IP_3_ signals were dependent on the histamine concentration in both S-cells and D-cells. The IP_3_ productions in S-cells were significantly smaller than those in D-cells under all three conditions, suggesting that the amount of IP_3_ production and/or IP_3_ dynamics should be involved in the formation of the cell-specific patterns of [Ca^2+^] changes. To understand the relationship between IP_3_ dynamics and Ca^2+^ dynamics more precisely, we investigated the changes in Ca^2+^ dynamics by modifying IP_3_ production in the following analyses.

### PLC Isozymes Expressed in HeLa Cells and their Specific Knockdown Using Small Interfering RNAs (siRNAs)

**Figure 3:**
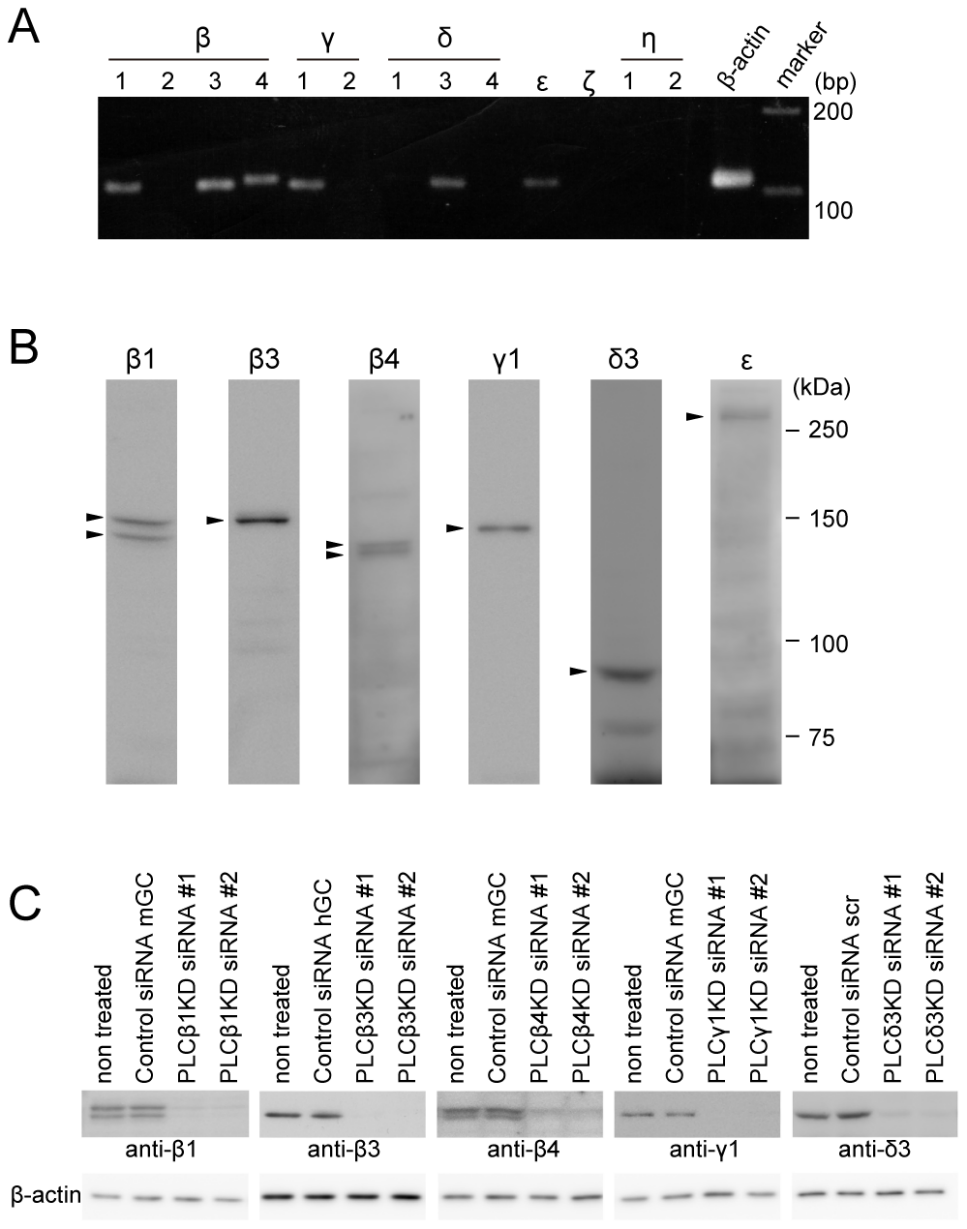
Identification of PLC isozymes expressed in HeLa cells and their specific knockdown using siRNAs. (A) Results of RT-PCR analyses using total RNA isolated from HeLa cells. (B) Western blotting analyses of endogenous PLC isozymes expressed in HeLa cells using isozyme-specific antibodies. Molecular mass markers are shown on the right. (C) Western blotting analyses of total lysates prepared from HeLa cells treated with PLC isozyme-specific siRNAs. The antibodies used are shown at the bottom. The results are representative of at least three independent experiments.

In a previous study [10], we found that there are multiple components of IP_3_ production in HeLa cells stimulated with histamine (see below). To identify the PLC isozymes involved in the generation of IP_3_ dynamics in histamine-stimulated HeLa cells, total RNA was isolated from HeLa cells and subjected to RT-PCR analyses with specific primer pairs (Table 1). We found that PLC-β1, -β3, -β4, -γ1, -δ3 and -ε were expressed at relatively high levels among the 13 PLC genes (Fig. 3A). All of the isozymes were successfully detected by western blotting with isozyme-specific antibodies (Fig. 3B). In the western blot analyses, two spliced variants of PLC-β1 and PLC-β4 were detected (Fig. 3B). Next, we examined the effects of specific knockdown of the PLC isozymes using siRNAs. As shown in Fig. 3C, the expressions of the endogenous PLC isozymes, except for PLC-ε, were markedly reduced by two different siRNAs for each isozyme. We confirmed that the PLC isozyme-specific knockdown did not affect the expression levels of the other PLC isozymes in HeLa cells (Fig. S3).

**Table 1:** Specific primer sequences for RT-PCR analyses.

Target gene	RT-PCR primer sequences^a^ (5′ to 3′)	Product size (bp)
PLC-β1	F TGTCTCAGCCCCTTTCTCAC	111
	R ACAGGAGCACTTGGCGATAC	
PLC-β2	F GAGGACTTCCCAGAACCTGTC	116
	R TCCTTCGTCATGTAGGGTTTG	
PLC-β3	F AAGTCCTTCGACCCCTTCAC	110
	R TAGATGCCCACCTTCCTGTC	
PLC-β4	F CCGAACTCGCATGGTTATG	114
	R TCATACACAGCTATTCTCAAGACAG	
PLC-γ1	F CGACTGGATTCCTCCAACTAC	108
	R GGCCTGGTTCATCTGCATAG	
PLC-γ2	F TCTGCGCTTTGTGGTTTATG	109
	R CAGAGGAACGGACCTGAATC	
PLC-δ1	F CCAGCAGCACTGAAGCCTAC	114
	R TGAAAGTATAGCCGTGGTAGATG	
PLC-δ3	F GGCCTATGTTAGGGCCTTTG	110
	R TCTTGGAGGTGAGGGTATGG	
PLC-δ4	F CTGTCGTTTACCACGGACAC	117
	R TCTCCAGGGACAAGATGACTG	
PLC-ε	F GAAAACACCAGCGATCTTCAG	107
	R TGCCTCTGGTTGTCCGATAG	
PLC-ζ	F TCGGTGCAGTTATATTCATGTG	117
	R TCTTCTCTGTGCGTGATAATTC	
PLC-η1	F GGAAGGGGAAGTTTCTGATG	112
	R CCTTATGAAAGATTCCACCTGATG	
PLC-η2	F GGACATCATCGAGCAGTTTG	119
	R GGTGCTCAGGGTTGAAGATG	
β-actin	F TCGTGCGTGACATTAAGGAG	110
	R GTCAGGCAGCTCGTAGCTCT	

a F and R denote foreword and reverse primers, respectively.

### Identification of PLC Isozymes Involved In Histamine-induced [IP_3_] Increases in HeLa Cells

**Figure 4:**
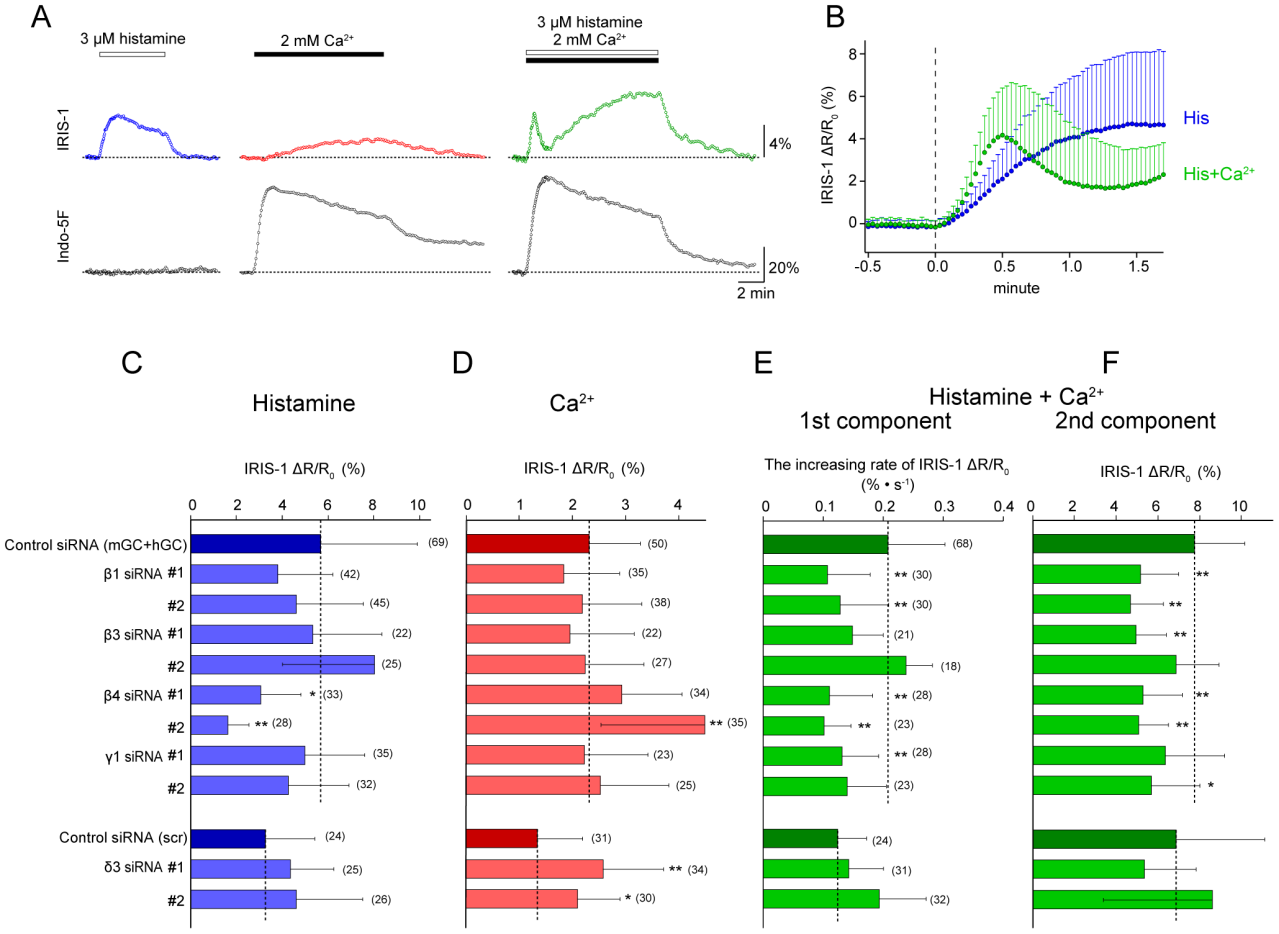
Identification of PLC isozymes involved in IP_3_ generation in HeLa cells stimulated with histamine. (A) Representative traces of IRIS-1 signal changes (ΔR/R_0_; top) and Indo-5F signal changes (F/F_0_; bottom) observed in thapsigargin-treated HeLa cells. The horizontal broken lines indicate the baseline levels. (B) Traces of the mean ± SD IRIS-1 signal changes (ΔR/R_0_) observed in thapsigargin-treated HeLa cells after addition of 3 µM histamine (blue circles) or 3 µM histamine plus 2 mM Ca^2+^ (green circles). (C–F) Effects of PLC isozyme knockdown on the IP_3_ increase evoked by 3 µM histamine alone (C), the IP_3_ increase evoked by 2 mM Ca^2+^ alone (D), the first component of the IP_3_ increase evoked by 3 µM histamine plus 2 mM Ca^2+^, and the second component of the IP_3_ increase evoked by 3 µM histamine plus 2 mM Ca^2+^. Data are shown as means ± SD. The numbers of cells measured are shown in parentheses. Statistical analyses were performed by one-way ANOVA followed by Scheffe’s multiple comparison test. *P<0.05, **P<0.01, vs. the values in control siRNA-transfected cells.

There are multiple components in the IP_3_ increases observed in HeLa cells [10]: (1) a fast IP_3_ increase in response to histamine receptor stimulation without cytosolic Ca^2+^ increase (Fig. 4A, left); (2) a slow IP_3_ increase in response to cytosolic Ca^2+^ increase alone (Fig. 4A, middle); and (3) a complex [IP_3_] change composed with a fast transient (1st component) and a slow gradual increase (2nd component) in response to the combination of histamine receptor stimulation and cytosolic Ca^2+^ increase (Fig. 4A, right). The presence of cytosolic Ca^2+^ enhanced the rate of the histamine-induced IP_3_ increase and also elicited a delayed negative feedback (Fig. 4B). We attempted to identify the PLC isozymes contributing to each component by measuring the IP_3_ dynamics in PLC isozyme-specific knockdown cells. To exclude the effects of Ca^2+^ released through IP_3_ receptor stimulation on the [IP_3_] changes, the cells were pretreated with thapsigargin to deplete intracellular Ca^2+^ stores, and cytosolic [Ca^2+^] was increased using the capacitive Ca^2+^ entry pathway activated by Ca^2+^ store depletion, using previously described methods [10]. We used the maximal IRIS-1 signal change or the increasing rate of the IRIS-1 signal as an index of PLC activity in the following experiments because these values were not affected by the IRIS-1 expression level, the resting level of [IP_3_], or the increasing rate of [Ca^2+^] or total amount of [Ca^2+^] increase mediated by capacitive Ca^2+^ entry (Fig. S4).

Figure 4C shows the results of PLC isozyme knockdown on the [IP_3_] increase in response to histamine receptor stimulation alone. Among the five isozymes examined, only PLC-β4 knockdown caused significant impairments of the [IP_3_] increase irrespective of the siRNA used. The maximal IRIS-1 change was significantly decreased by 46% (siRNA#1) and 71% (siRNA#2) compared with the mean change with the control siRNAs mGC and hGC (Fig. 4C). These findings indicate that PLC-β4 is selectively activated downstream of histamine receptors in the absence of [Ca^2+^] elevation in HeLa cells.

Figure 4D shows the results of PLC isozyme knockdown on the [IP_3_] increase in response to cytosolic Ca^2+^ increase alone, which was elicited by the application of extracellular 2 mM CaCl_2_ whithout histamine. In thapsigargin-treated HeLa cells, store-operated Ca^2+^ influx channels on the plasma membrane are activated by the depletion of intracellular Ca^2+^ stores and the addition of extracellular Ca^2+^ immediately elicites [Ca^2+^] increases through the Ca^2+^ influx channels [10]. Because knockdown of any single PLC isozyme alone did not significantly decrease the amount of the IP_3_ increase (Fig. 4D), multiple PLC isozymes might be activated in response to [Ca^2+^] elevation. A significant increase in [IP_3_] was observed in cells treated with PLCβ4KD siRNA#2 (Fig. 4D), even though the treatment with this siRNA did not change the expression levels of other PLC isozymes (Fig. S3). Because the other siRNA designed for PLC-β4, PLCβ4KD siRNA#1, did not increase [IP_3_] significantly (Fig. 4D), the increase in [IP_3_] may be caused by some side-effects of PLCβ4KD siRNA#2. PLC-δ3 knockdown cells using two different siRNAs showed significant increases in [IP_3_] evoked by Ca^2+^ increase (Fig. 4D). The treatments with these two siRNAs did not change the expression level of other PLC isozymes (Fig. S3). PLC-δ3 may possess some inhibitory effects on the Ca^2+^-dependent IP_3_ production in HeLa cells.

Figure 4E (1st component) and 4F (2nd component) show the results of PLC isozyme knockdown on the [IP_3_] increases in response to the combination of histamine receptor stimulation and Ca^2+^ increase. Both the 1st and 2nd components of the [IP_3_] increase after addition of 3 µM histamine plus 2 mM Ca^2+^ were impaired by knockdown of PLC-β1 or PLC-β4. The increasing rate of the IRIS-1 signal of the 1st component was significantly reduced by 48% (PLCβ1KD siRNA#1), 38% (PLCβ1KD siRNA#2), 47% (PLCβ4KD siRNA#1), and 51% (PLCβ4KD siRNA#2) compared with the mean value for the control siRNAs mGC and hGC (Fig. 4E). The maximal IRIS-1 signal change of the 2nd component was significantly reduced by 33% (PLCβ1KD siRNA#1), 40% (PLCβ1KD siRNA#2), 32% (PLCβ4KD siRNA#1), and 34% (PLCβ4KD siRNA#2) compared with the mean value for the control siRNAs mGC and hGC (Fig. 4F). These findings indicate that both PLC-β1 and PLC-β4 are activated by histamine stimulation accompanied by [Ca^2+^] elevation and contribute to the generation of the 1st and 2nd components. Delayed inhibition of IP_3_ production after the 1st component of the [IP_3_] increase was also detected in PLC-β1 and PLC-β4 knockdown cells (Fig. S5), suggesting that this inhibitory effect was mediated by components upstream of PLC (GPCR and/or G proteins) or by both PLC-β1 and PLC-β4 to the same degree.

### Roles of PLC-β1 and PLC-β4 in the Generation of Ca^2+^ Oscillations

**Figure 5:**
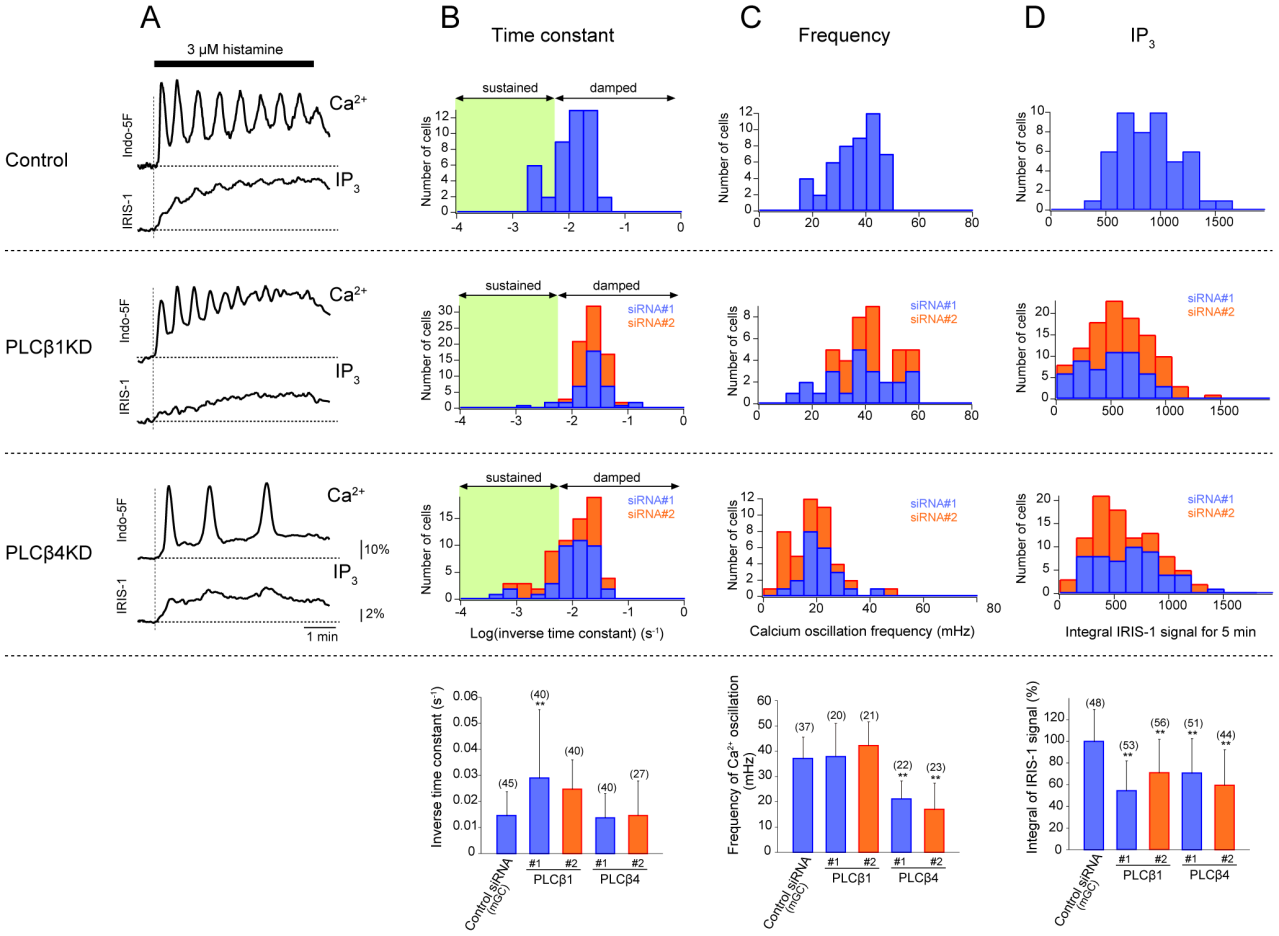
Effects of PLC-β1 or PLC-β4 knockdown on Ca^2+^ and IP_3_ dynamics evoked by histamine stimulation. (A) Representative traces of Indo-5F signal changes (F/F_0_; top) and IRIS-1 signal changes (ΔR/R_0_; bottom) in siRNA-treated cells stimulated with 3 µM histamine. The siRNAs used are shown on the left. The horizontal broken lines indicate the baseline levels of the IRIS-1 and Indo-5F signals. The vertical broken lines indicate the onsets of stimulation. Stacked histograms of the inverse time constants for exponential decay of the Ca^2+^ oscillation amplitude (B), Ca^2+^ oscillation frequencies (C), and integrated IP_3_ signals (D) of cells treated with control siRNA (top row), PLCβ1KD siRNAs (second row), and PLCβ4KD siRNAs (third row). The results for the two siRNAs are shown in different colors. The means ± SD are shown at the bottom. The numbers of cells measured are shown in parentheses. Statistical analyses were performed by one-way ANOVA followed by Scheffe’s multiple comparison test. **P<0.01, vs. the values in control siRNA mGC-transfected cells.

We examined how PLC-β1 and PLC-β4 contribute to the generation of Ca^2+^ dynamics by measuring [Ca^2+^] in PLC-β1 and PLC-β4 knockdown cells stimulated with 3 µM histamine. We found that specific knockdown of PLC-β1 resulted in alterations of both Ca^2+^ dynamics and IP_3_ dynamics (Fig. 5A). One of the obvious differences was that sustained Ca^2+^ oscillations were hardly detected in PLC-β1 knockdown cells (Fig. 5B). The mean inverse time constants of Ca^2+^ spike amplitude decay (0.0290±0.0263 s^−1^ for siRNA#1 and 0.0247±0.0114 s^−1^ for siRNA#2) were larger than that in control cells (0.0146±0.0093 s^−1^, *n* = 45) and the fraction of cells showing damped oscillations was increased (Fig. 5B). In contrast, the mean Ca^2+^ oscillation frequencies in PLC-β1 knockdown cells were similar to that in control cells (37.1±8.6 mHz) (Fig. 5C). The integrals of the IP_3_ signals were significantly reduced in PLC-β1 knockdown cells (Fig. 5D).

When the expression of PLC-β4 was selectively reduced, different phenotypes were observed (Fig. 5A). In PLC-β4 knockdown cells, sustained Ca^2+^ oscillations with small inverse time constants (less than 10^−3^ s^−1^) were detected in some cells, but the overall mean inverse time constants did not differ significantly from that in control cells (Fig. 5A and B). The mean frequencies of Ca^2+^ oscillations in PLC-β4 knockdown cells (21.1±7.2 mHz for siRNA#1 and 17.0±10.5 mHz for siRNA#2) were significantly smaller than that in control cells (37.1±8.6 mHz) (Fig. 5C). The integrals of the IP_3_ signals were also reduced (Fig. 5D), similar to the case for PLC-β1 knockdown cells. These findings indicate that knockdown of PLC-β1 increases the time constant of Ca^2+^ spike amplitude decay, while knockdown of PLC-β4 decreases the Ca^2+^ oscillation frequency in HeLa cells stimulated with histamine. It is important to note that the integrals of the IP_3_ signals were similarly reduced in both PLC-β1 and PLC-β4 knockdown cells (Fig. 5D), while the temporal patterns of IP_3_ dynamics were characteristically different between these cells (Fig. 5A). In PLC-β1 knockdown cells, fluctuation of the IP_3_ signals was hardly detected during Ca^2+^ oscillations, while fluctuations of [IP_3_] synchronized with Ca^2+^ spikes were obviously detected in PLC-β4 knockdown cells (Fig. 5A). These findings suggest that the temporal pattern of IP_3_ dynamics, but not the absolute IP_3_ concentration, is important for determining the pattern of [Ca^2+^] changes, such as sustained oscillations and damped oscillations.

**Figure 6:**
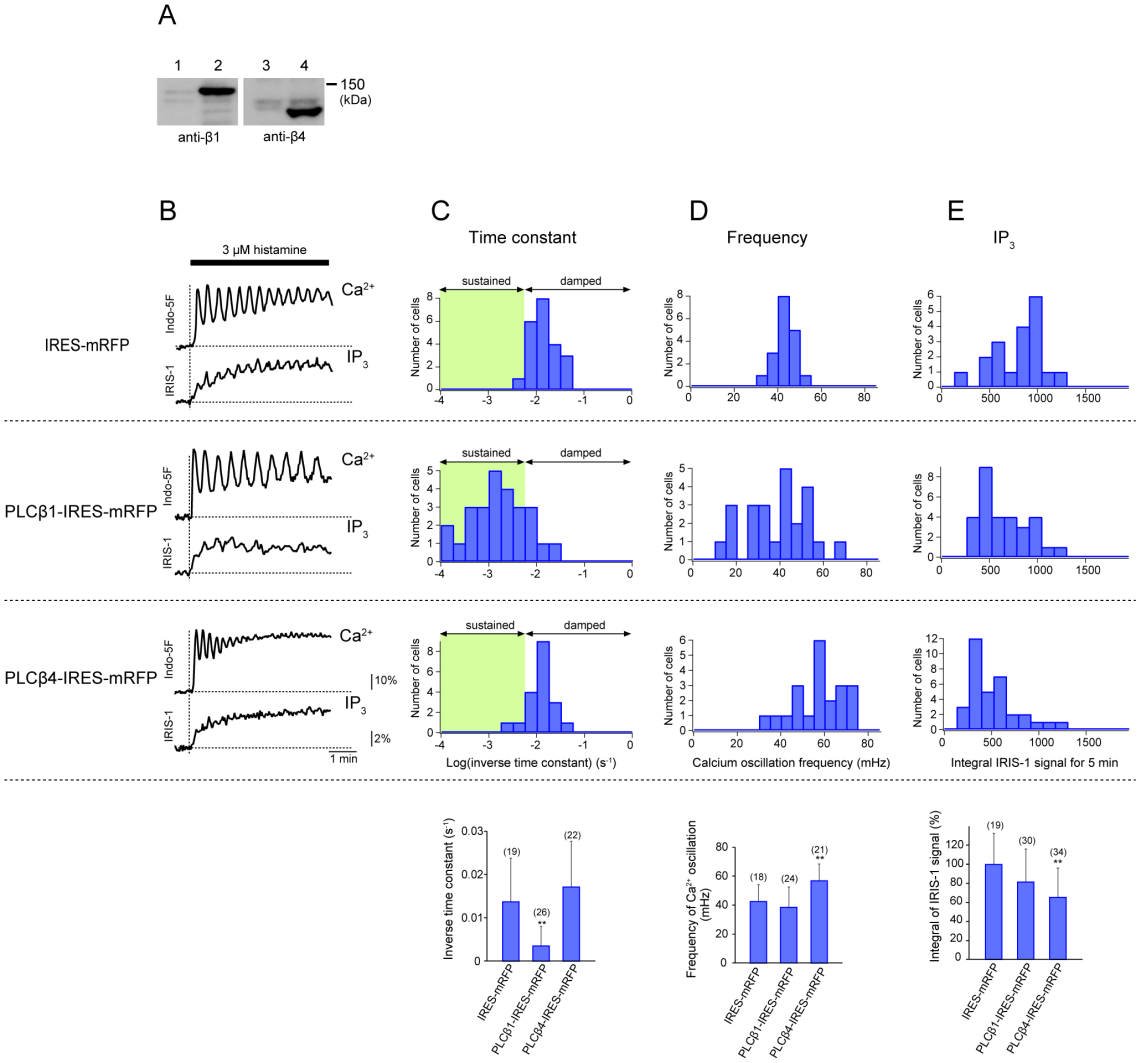
Effects of PLC-β1 or PLC-β4 overexpression on Ca^2+^ and IP_3_ dynamics evoked by histamine stimulation. (A) Western blotting analyses of cell lysates prepared from HeLa cells transfected with PLC-β1-IRES-mRFP (lane 2) or PLC-β4-IRES-mRFP (lane 4). Non-transfected cells were used as controls (lanes 1 and 3). (B) Representative traces of Indo-5F signal changes (F/F_0_; top) and IRIS-1 signal changes (ΔR/R_0_; bottom) in transfected cells stimulated with 3 µM histamine. The plasmid DNAs used to transfect the cells are shown on the left. The horizontal broken lines indicate the baseline levels of IRIS-1 and Indo-5F signals. The vertical broken lines indicate the onsets of stimulation. (B–D) Histograms for the inverse time constants for exponential decay of the Ca^2+^ oscillation amplitude (B), Ca^2+^ oscillation frequencies (C), and integrated IP_3_ signals (D) in cells expressing mRFP (top row), PLC-β1 and mRFP (second row), and PLC-β4 and mRFP (third row). The means ± SD are shown at the bottom. The numbers of cells measured are shown in parentheses. Statistical analyses were performed by one-way ANOVA followed by Scheffe’s multiple comparison test. **P<0.01, vs. the values in IRES-mRFP transfected cells.

To confirm the effects of PLC-β1 or PLC-β4 knockdown on the generation of Ca^2+^ dynamics, we examined cells transfected with PLCβ1- or PLCβ4-IRES-mRFP constructs (Fig. 6A) and subjected mRFP-positive cells to single-cell imaging of [IP_3_] and [Ca^2+^]. Overexpression of PLC-β1 or PLC-β4 led to different patterns of Ca^2+^ dynamics (Fig. 6B). PLC-β1-overexpressing cells showed sustained Ca^2+^ oscillations more frequently than control cells transfected with the IRES-mRFP construct (Fig. 6B), and the mean inverse time constant of Ca^2+^ spike amplitude decay (0.00349±0.00456 s^−1^, *n* = 26) was significantly smaller than that in control cells (0.0137±0.0100 s^−1^, *n* = 19) (Fig. 6C). In contrast, PLC-β4-overexpressing cells showed Ca^2+^ oscillations with higher frequency (mean ± SD, 56.8±11.6 mHz) than that in control cells (mean ± SD, 42.5±4.5 mHz) (Fig. 6D). These findings are consistent with those in the RNAi-mediated silencing experiments when we make two assumptions as follows. First, histamine-induced IP_3_ production is mainly operated by PLC-β1 and PLC-β4 in HeLa cells. Therefore, PLC-β4 alone works in PLC-β1 knockdown cells and PLC-β1 alone works in PLC-β4 knockdown cells. No compensation by the other PLC isozymes exists in knockdown cells, as supported by the data shown in Fig. S3. Second, overexpressed PLC-β isozyme has a dominant activity, even though the other type of endogenous PLC-β is expressed in the transfected cells. According to these assumptions, we can conclude that PLC-β1 activity is involved in determination of the time-dependence of Ca^2+^ spike amplitude decay, while PLC-β4 activity is involved in determination of the Ca^2+^ oscillation frequency in HeLa cells. As previously described in some reports [16], [17], the overexpression of PLC-βs alone without G proteins did not cause a marked increase in the IP_3_ production (Fig. 6E). Instead, the integral of the IP_3_ signals was significantly decreased in PLC-β4-overexpressing cells to 65% of the value in control cells suggesting that an excess amount of PLC-β4 would function inhibitory for the IP_3_ production. The exact mechanism of this phenomenon is not clear, but one possibility is that GTP hydrolysis by Gα_q_ would be promoted due to an excess amount of PLC-β4 because PLC-βs are GTPase activating proteins for Gα_q_ [12]. When we compared cells showing almost the same integral amounts of IP_3_ production, a phenotypic difference in the patterns of [Ca^2+^] changes was still observed between PLC-β1-overexpressing cells and PLC-β4-overexpressing cells (Fig. 6B). Taken together, these findings provide further support for the notions that a constant threshold of [IP_3_] for driving Ca^2+^ spikes does not exist, and that a temporal pattern of IP_3_ dynamics is critical for the determination of Ca^2+^ signaling patterns.

## Discussion

During the past decade, it has become increasingly evident that genetically identical cells can exhibit variability in their cellular responses, even in identical environments. Underlying much of this variability is the stochasticity in gene expression that can produce unique proteomes in individual cells, and this cell population heterogeneity is an important factor in processes ranging from stem cell differentiation to chemotherapy resistance [18], [19]. In the present study, we found that individual HeLa cells exhibited characteristic and highly reproducible patterns of Ca^2+^ changes in response to bath applications of histamine, even in the same microscopic field of view, as reported in other cell lines [5], [13]–[15]. We quantified the patterns of Ca^2+^ signals by the Ca^2+^ oscillation frequencies and the time constants of Ca^2+^ spike amplitude decay, and found that a difference in histamine sensitivity could not account for the cell-to-cell variability in Ca^2+^ signals. A remarkable finding in this study is that the IP_3_ dynamics are characteristically different depending on the patterns of Ca^2+^ signals (Fig. 1), suggesting that the cell-to-cell variety in Ca^2+^ signals originates from the upstream of the IP_3_ generation process in the signal transduction cascade in this cell type.

### Identification of PLC Isozymes Activated in HeLa Cells Stimulated with Histamine

HeLa cells, an epithelial cell line derived from a carcinoma of the human uterine cervix, express the histamine H_1_ receptor [20]. Ten Gα proteins among the 16 mammalian Gα proteins and four Gβ proteins among five Gβ proteins have been detected in HeLa cells [21]. RT-PCR analyses positively identified transcripts for all 12 Gγ genes in HeLa cells [21]. In this study, we found that, among the 13 PLC isozyme genes, six (PLC-β1, -β3, -β4, -γ1, -δ3, and -ε) are expressed in HeLa cells. RNAi-mediated silencing of the expressions of the PLC genes showed that at least two isozymes in the PLC-β family, PLC-β1 and PLC-β4, are actually involved in the generation of IP_3_ production evoked by histamine stimulation. Among the three PLC-β isozymes identified in HeLa cells, specific knockdown of PLC-β3 did not induce a significant decrease in the histamine-induced IP_3_ production. Recently, Adjobo-Hermans et al. showed that PLC-β1 and PLC-β4 are enriched at the plasma membrane [22], while PLC-β2 and PLC-β3 are mainly localized in the cytosol in resting HeLa cells following expression of GFP-fused PLC-β constructs. Our findings suggest that the intracellular localization of PLC-β isozymes is a critical factor for the selection of PLC-β isozymes activated downstream of the G protein-coupled histamine H_1_ receptor.

### A Possible Mechanism that Underlies the Generation of Cell-specific Patterns of Histamine-induced Ca^2+^ Signals in HeLa Cells

**Figure 7:**
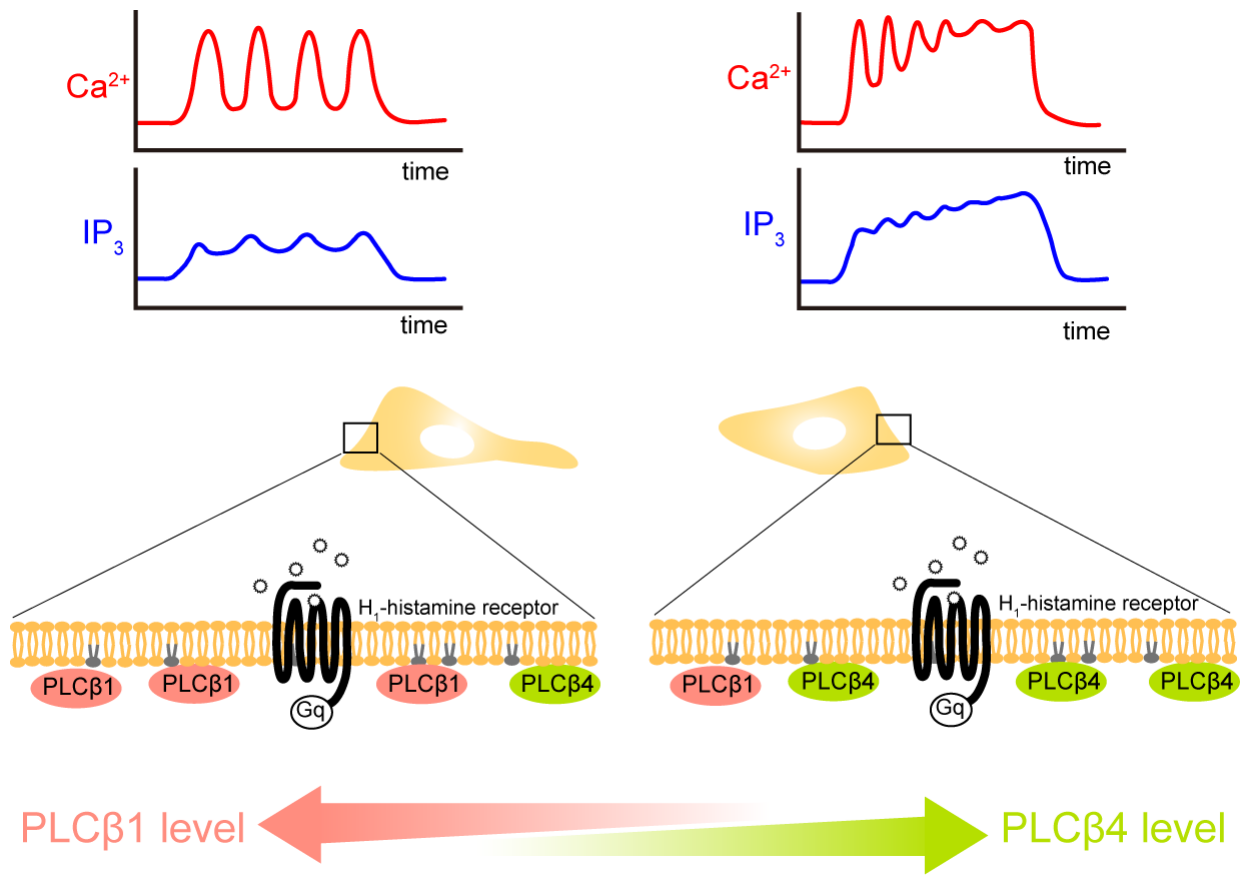
A possible mechanism that underlies the generation of cell-specific patterns of histamine-induced Ca^2+^ signals. The diagrams show the typical IP_3_ and Ca^2+^ dynamics observed in HeLa cells stimulated with histamine. Low-frequency sustained Ca^2+^ oscillations tend to be observed in cells in which PLC-β1 is dominant (left), while high-frequency damped oscillations tend to be observed in cells in which PLC-β4 is dominant (right).

The RNAi-mediated silencing experiments and PLC isozyme overexpression experiments provide coherent evidence that PLC-β1 activity is involved in determination of the time constant of Ca^2+^ spike amplitude decay during Ca^2+^ oscillations, while PLC-β4 activity is involved in determination of the Ca^2+^ oscillation frequency in HeLa cells stimulated with histamine. These findings suggest that the relative amounts of PLC-β1 and PLC-β4 have a large impact on the pattern of histamine-induced Ca^2+^ signals. We hypothesize that stochastic variations in the amounts of PLC-β1 and PLC-β4 expressed in HeLa cells are involved in the generation of the non-genetic cell-to-cell variability in agonist-induced Ca^2+^ signals (Fig. 7). According to this hypothesis, low-frequency sustained Ca^2+^ oscillations tended to be observed in cells in which PLC-β1 was dominant, while high-frequency damped oscillations tended to be observed in cells in which PLC-β4 was dominant (Fig. 7). The former may correspond to the S-cells shown in Fig. 2A, while the latter may correspond to the D-cells shown in Fig. 2B. Ambler et al. described that sister cells in a BC3H-1 clonal cell population displayed unique temporal responses in histamine-induced Ca^2+^ signals immediately following cell division [13], and proposed that the linkage of receptor occupancy to Ca^2+^ elevation is a functionally unique property for individual cells that can be influenced by epigenetic factors. Quantification of the individual PLC isozymes and rapid manipulation of their expression and/or intracellular localization in single HeLa cells will be useful to verify our hypothesis in future studies.

### Regulatory Factors for PLC-β Activity

Comparisons of the functional differences between PLC-β1 and PLC-β4 should provide clues toward understanding the mechanistic basis for the generation of cell-specific patterns of Ca^2+^ signals. The RNAi-mediated silencing experiments revealed that PLC-β1 only became activated when the histamine stimulation was accompanied by [Ca^2+^] increases in thapsigargin-treated cells, whereas PLC-β4 was activated by histamine stimulation with or without [Ca^2+^] elevation. These findings are consistent with previous biochemical measurements showing that purified PLC-β1 exhibits Ca^2+^-dependent PIP_2_ hydrolysis activity with a maximal activation at 100–220 nM [Ca^2+^] [23], while the activity of purified PLC-β4 is less sensitive to Ca^2+^ and the PLC-β4 activity in the absence of Ca^2+^ is higher than those of the other PLC-βs [24]. Because feedback regulation by cytosolic Ca^2+^ must be involved in the Ca^2+^ signal formation, the difference in Ca^2+^ sensitivity for the PIP_2_ hydrolysis activity between PLC-β1 and PLC-β4 should be critical in determining the pattern of cell-specific Ca^2+^ signals.

PLC-β isozymes are regulated by heterotrimeric G proteins, whose activities are controlled by the balance of the rate of GTP binding, which causes activation, and the rate of GTP hydrolysis, which terminates activation [25], [26]. Because Gα subunits show remarkably slow rates of basal guanine nucleotide exchange and a low rate of GTP hydrolysis [27], [28], the GTPase catalytic cycle is regulated by GPCRs, which accelerate GTP binding, and by GTPase-activating proteins (GAPs), which accelerate hydrolysis. PLC-βs are GAPs for Gα_q_ [29] and accelerate the deactivation limb of the cycle. In the continued presence of an agonist, it has been proposed that activated GPCR stays bound to Gα_q_ through multiple cycles of GTP binding and hydrolysis because the rate of dissociation of the GPCR from GTP-bound Gα_q_ is much slower than that of the PLC-β-promoted return of the G protein to the GDP-bound state. This phenomenon has been referred to as a kinetic scaffold [27], [28], [30], and the GAP activities of the PLC-β isozymes may influence the temporal pattern of the activation of each isozyme independently. Recently, it was shown that the intracellular localization of PLC-β1 is regulated by PKC phosphorylation [31] and by translin-associated factor X binding [32]. In addition to the PIP_2_ hydrolysis activity, examination of the differences between PLC-β1 and PLC-β4 in terms of these regulatory factors in living cells will be pivotal for understanding the molecular basis of their differential roles in the generation of Ca^2+^ signals.

## Materials and Methods

### Materials

Anti-PLC-β1, anti-PLC-β3, anti-PLC-β4, and anti-PLC-γ1 rabbit polyclonal antibodies were obtained from Santa Cruz Biotechnology Inc. A mouse monoclonal antibody against β-actin was purchased from Sigma-Aldrich. A mouse monoclonal antibody against PLC-δ3 [33] was used. Mouse PLC-β1 cDNA (U85712) and mouse PLC-β4 cDNA (NM_013829) were subcloned and inserted into pBluescriptII KS(+). A rabbit polyclonal antibody against PLC-ε [34] was a generous gift from Dr. T. Kataoka (Kobe University, Kobe, Japan). Two different predesigned siRNA duplexes targeting each of PLC-β1, PLC-β3, PLC-β4, PLC-γ1, and PLC-ε (Stealth™ RNAi) were purchased from Invitrogen. Two Stealth™ RNAi Negative Control Medium GC Duplexes (mGCs) and two Stealth™ RNAi Negative Control High GC Duplexes (hGCs) (both from Invitrogen) were used as negative controls. For knockdown of PLC-δ3, two original siRNAs were synthesized (ST Pharm Co. Ltd.). The sequence of PLCδ3KD siRNA#1 (Table 2) was scrambled to minimize the sequence homology with any known vertebrate transcripts and used to synthesize a negative control siRNA. The nucleotide sequences of the siRNAs used in this study are shown in Table 2.

**Table 2:** siRNA sequences.

Name	Sequences (5′ to 3′)	GC%^a^	Position^b^
Control siRNA mGC	GGUAGGUGAGUGUACAGACGCAAUA	48	
Control siRNA mGC	CAGGACGUCUAGCUGUAGUCGCCAU	56	
PLCβ1KD siRNA #1	GCUACUGGAUCUCAGCCUUGUCAAA	48	186
PLCβ1KD siRNA #2	GCCCUCGACCUGAAAUUGAUAACAU	44	659
PLCβ3KD siRNA #1	GGCUUCACUUCGCAUUGCAGCCUUU	52	2372
PLCβ3KD siRNA #2	ACCCGAGACUCAACGAAGUGCUGUA	52	766
PLCβ4KD siRNA #1	GCUGCCAGAUGGUUUCACUGAACUA	48	1928
PLCβ4KD siRNA #2	GCAUGGUUAUGAAUAAUGGACUCAA	36	2222
PLCγ1KD siRNA #1	CCCUGCGCUGUAAUGAGUUUGAGAU	48	1928
PLCγ1KD siRNA #2	CCUUGUUGACCUCAUCAGCUACUAU	44	2199
PLCεKD siRNA #1	CGCCACCCUCCAAAGGACUUCAAUA	52	1362
PLCεKD siRNA #2	CAGCGAUCUUCAGCCUGACCUAGAU	52	3792
Control siRNA scr	GGCAUAGCCAGAUGGUAGAGAUAAGAA	44	
PLCδ3KD siRNA #1	AGAUGAGCUUCAAGGAGAUCAAGAGAG	44	602
PLCδ3KD siRNA #2	UGGUCAACGUGGACAUGAACGACAUAG	48	638

a The GC% column indicates the GC content of each siRNA sequence.

b The numbers in the position column denote where the sequence is located in the mRNA of the target gene (counted from the first nucleotide of the start codon) of human origin.

### Plasmid Construction

The plasmid IRES-mRFP was prepared from IRES2-AcGFP1 (Clontech Laboratories Inc.) by replacing the AcGFP1 cDNA with an mRFP cDNA. A full-length mouse PLC-β1 cDNA was obtained by PCR from mouse PLCβ1-pBluescriptII KS(+), and cloned into the EcoRI and BamHI sites of IRES-mRFP. A full-length mouse PLC-β4 cDNA was obtained from mouse PLCβ4-pBluescriptII KS(+) by XhoI digestion, and inserted into IRES-mRFP.

### Cell Culture

HeLa cells were maintained in DMEM (Nacalai Tesque) supplemented with 10% FBS at 37°C under 5% CO_2_.

### RT-PCR

Total RNA was extracted from HeLa cells using a TRIzol Reagent Kit (Invitrogen), and reverse-transcribed using random primers and SuperScriptII reverse transcriptase (Invitrogen) according to the manufacturer’s protocol. cDNA fragments for the 13 PLC genes were amplified by PCR using specific primer sets (Table 1). The primers were designed using Primer 3 software to fullfil the following requirements: (1) the PCR products should contain an exon–exon junction to prevent amplification of any contaminating genomic DNA; (2) all known splicing variants should be detected; (3) the PCR products should be approximately 100 bp; and (4) the primers should have almost the same T_m_ values to maintain uniform amplification efficiency. Primers for β-actin were designed under similar criteria and used as an internal control. The PCR fragments were analyzed by 2% agarose gel electrophoresis.

### Western Blotting

Total HeLa cell lysates were prepared by direct lysis with SDS sample buffer (62.5 mM Tris-HCl pH 6.8, 10% glycerol, 2% SDS, 5% β-mercaptoethanol, 0.05% bromophenol blue) and subjected to electrophoresis. The separated proteins were transferred onto PVDF membranes (Millipore), and the PLC isozymes and β-actin were probed with specific primary antibodies. After treatment with HRP-conjugated anti-rabbit or anti-mouse secondary antibodies (GE Healthcare), the antibody-bound proteins were detected with chemiluminescence reagents (ECL: GE Healthcare) and a LAS4000mini system (Fujifilm).

### siRNA Transfection

Fresh culture medium (0.8 ml) was supplied to HeLa cells cultured in 35-mm dishes at 30 min prior to transfection. Next, 1 µl of 20 µM siRNA solution and 1 µl of LipofectAMINE 2000 (Invitrogen) were independently diluted in 100 µl of Opti-MEM (Invitrogen), and the two mixtures were combined and incubated for 20 min at room temperature. The mixed solution was added to the cells to give a final concentration of 20 nM siRNA. After incubation for 8–12 h in a CO_2_ chamber, the siRNA solution was removed by replacement with 1.5 ml of fresh culture medium.

### Imaging

For imaging of siRNA-treated cells, HeLa cells at 36–42 h after siRNA transfection were transfected with plasmids containing the IRIS-1 cDNA using a transfection reagent (TransIT; Mirus). The cells were subjected to imaging at 24–30 h after the IRIS-1 transfection. For imaging of cells expressing recombinant PLC-βs, HeLa cells were transfected with PLCβ1-IRES-mRFP, PLCβ4-IRES-mRFP, or IRES-mRFP plasmids using the TransIT transfection reagent at 20–36 h before IRIS-1 transfection. After loading the cells with 5 µM Indo-5F AM (Dojindo), imaging was performed under a constant flow (2 ml/min) of balanced salt solution (20 mM Hepes pH 7.4, 115 mM NaCl, 5.4 mM KCl, 1 mM MgCl_2_, 2 mM CaCl_2_, 10 mM glucose) as an imaging medium at 37°C. For experiments with artificial control of [Ca^2+^], cells were pretreated with 1 µM thapsigargin in the absence of extracellular Ca^2+^ to deplete the cytosolic Ca^2+^ stores by blocking sarcoplasmic or endoplasmic reticulum Ca^2+^-ATPase pumps. Imaging was performed at 37°C using an inverted microscope (IX-71; Olympus) with a cooled charge-coupled device camera (ORCA-ER; Hamamatsu Photonics) and a 40× (NA 1.35) objective. A 330–348-nm excitation filter and 460–510-nm emission filter and a 425–445-nm excitation filter and a pair of 460–510-nm (cyan) and 525–565-nm (yellow) emission filters were used for the fluorochromes Indo-5F and IRIS-1, respectively. An emission splitter (W-view; Hamamatsu Photonics) was used with a fast light source exchanger (DG-4; Sutter Instrument Co.). Sequential excitation of IRIS-1 and Indo-5F was performed using a 450-nm dichroic mirror and two excitation filters (425–445-nm filter for IRIS-1 and 330–348-nm filter for Indo-5F). Dual emission at 460–490 nm (for IRIS-1 and Indo-5F) and >520 nm (for IRIS-1) was split with a 460–490-nm filter, a long-path 520-nm barrier filter, and two 505-nm dichroic mirrors equipped in W-view. Images were acquired at 0.5 or 1 Hz for measurements of Ca^2+^ oscillations in intact cells and at 0.17, 0.25, or 0.5 Hz for measurements in thapsigargin-treated cells. Image acquisition was performed with MetaFluor software (Molecular Devices). Spectral analyses of Ca^2+^ oscillations were performed as described previously [35].

## Supporting Information

Figure S1 **Histogram of the time constants for exponential decay of the Ca^2+^ spike amplitude.** (A) Ca^2+^ spike amplitudes were defined as shown in the left scheme. The time constants were estimated by fitting the amplitudes with a single exponential function, as shown on the right. (B) Histogram of the time constants of Ca^2+^ spike amplitude decay observed in HeLa cells stimulated with 3 µM histamine. The arrow indicates the threshold for dividing the cells into S-cells and D-cells.
(PDF)

Figure S2 **Difference in histamine concentration-dependence of the amplitude of the first Ca^2+^ spike and the IP_3_ production between S-cells and D-cells.** (A) Relationships between the histamine concentrations and the peak amplitudes of the first increase in Indo-5F signals (F/F_0_) in S-cells (red) and D-cells (blue). (B) Mean values of the Indo-5F signal changes shown in (A). Statistical analyses were performed by one-way ANOVA followed by Scheffe’s multiple comparison test. *P<0.05, **P<0.01. NS: not significant. (C) Relationships between the histamine concentration and the integrated IP_3_ signals observed in S-cells (red) and D-cells (blue). Data represent means ± SD. Statistical analyses were performed using Student’s *t*-test. *P<0.05.
(PDF)

Figure S3 **PLC isozyme-specific knockdown does not affect the expressions of other PLC isozymes.** Western blotting analyses of total lysates prepared from HeLa cells treated with PLC isozyme-specific siRNAs. The isozyme-specific antibodies used for the western blotting analyses are shown on the left. The data are representative of at least two independent experiments.
(PDF)

Figure S4 **Validation of the IRIS-1 signal changes observed in thapsigargin-treated HeLa cells.** (A and B) The maximal IRIS-1 signal changes after addition of 3 µM histamine (A) and 2 mM Ca^2+^ (B) were plotted against the basal CFP intensity of IRIS-1 (top), resting IRIS-1 C/Y ratio (middle), and integrated value of Indo-5F change for 10 min (bottom). (C) The initial rate of IRIS-1 signal change (1st component) after addition of 3 µM histamine plus 2 mM Ca^2+^ was plotted against the basal CFP intensity of IRIS-1 (top), resting IRIS-1 C/Y ratio (middle), and initial rate of Indo-5F change (bottom). (D) The maximal IRIS-1 signal changes after addition of 3 µM histamine plus 2 mM Ca^2+^ (2nd component) were plotted against the basal CFP intensity of IRIS-1 (top), resting IRIS-1 C/Y ratio (middle), and integrated value of Indo-5F change for 10 min (bottom).
(PDF)

Figure S5 **[IP_3_] changes after histamine stimulation plus [Ca^2+^] elevation in thapsigargin-treated PLC-β1 and PLC-β4 knockdown cells.** Representative traces of IRIS-1 signal changes (ΔR/R_0_) after addition of 3 µM histamine (horizontal open bars) plus 2 mM Ca^2+^ (horizontal filled bars) observed in thapsigargin-treated HeLa cells transfected with control siRNA mGC (left), PLCβ1KD siRNAs (middle), or PLCβ4KD siRNAs (bottom).
(PDF)
