# Supplementary figures and images for: Phospholipase C-β1 and β4 Contribute to Non-Genetic Cell-to-Cell Variability in Histamine-Induced Calcium Signals in HeLa Cells
Source: PLoS One. 2014 Jan 27;9(1):e86410. doi: 10.1371/journal.pone.0086410 (PMC3903530; doi:10.1371/journal.pone.0086410)

A

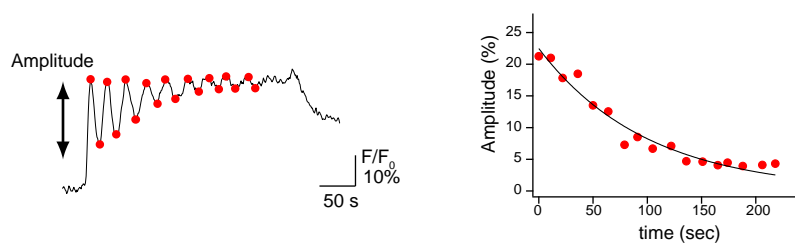

B

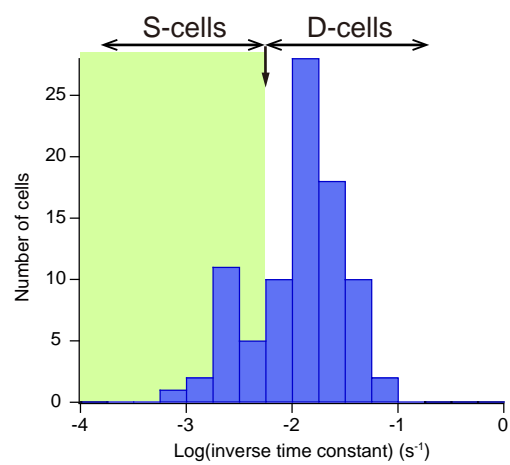

Figure S1

Supplement: Figure S1 — Histogram of the time constants for exponential decay of the Ca2+ spike amplitude. (A) Ca2+ spike amplitudes were defined as shown in the left scheme. The time constants were estimated by fitting the amplitudes with a single exponential function, as shown on the right. (B) Histogram of the time constants of Ca2+ spike amplitude decay observed in HeLa cells stimulated with 3 µM histamine. The arrow indicates the threshold for dividing the cells into S-cells and D-cells. (PDF) [file pone.0086410.s001.pdf]

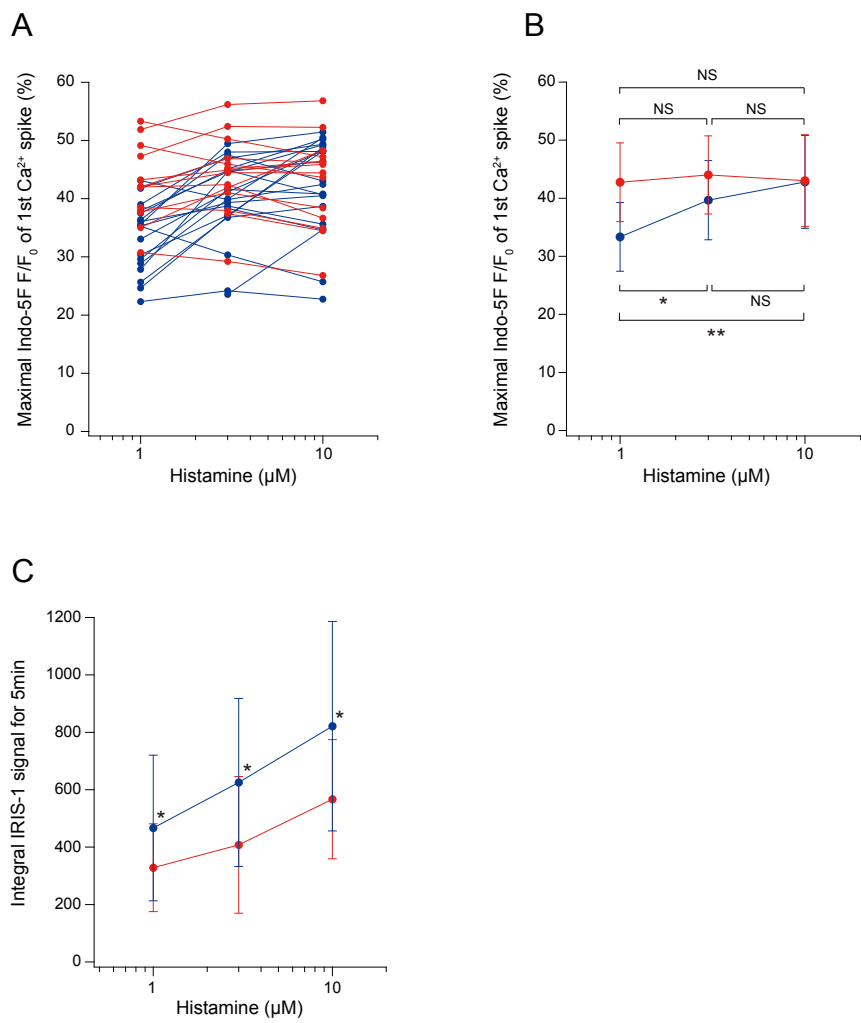

Figure S2

Supplement: Figure S2 — Difference in histamine concentration-dependence of the amplitude of the first Ca2+ spike and the IP3 production between S-cells and D-cells. (A) Relationships between the histamine concentrations and the peak amplitudes of the first increase in Indo-5F signals (F/F0) in S-cells (red) and D-cells (blue). (B) Mean values of the Indo-5F signal changes shown in (A). Statistical analyses were performed by one-way ANOVA followed by Scheffe’s multiple comparison test. *P<0.05, **P<0.01. NS: not significant. (C) Relationships between the histamine concentration and the integrated IP3 signals observed in S-cells (red) and D-cells (blue). Data represent means ± SD. Statistical analyses were performed using Student’s t-test. *P<0.05. (PDF) [file pone.0086410.s002.pdf]

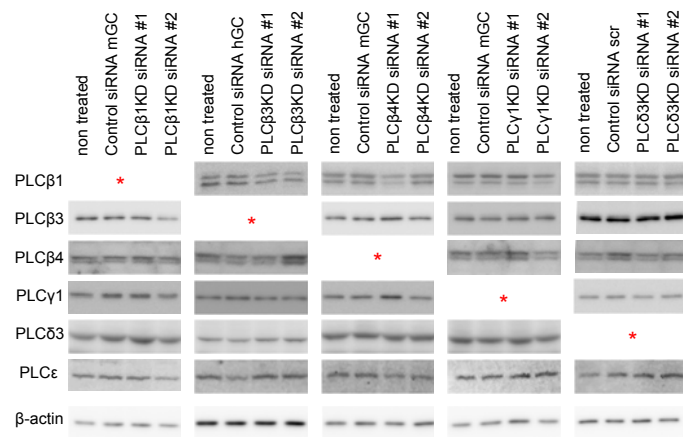

Figure S3

Supplement: Figure S3 — PLC isozyme-specific knockdown does not affect the expressions of other PLC isozymes. Western blotting analyses of total lysates prepared from HeLa cells treated with PLC isozyme-specific siRNAs. The isozyme-specific antibodies used for the western blotting analyses are shown on the left. The data are representative of at least two independent experiments. (PDF) [file pone.0086410.s003.pdf]

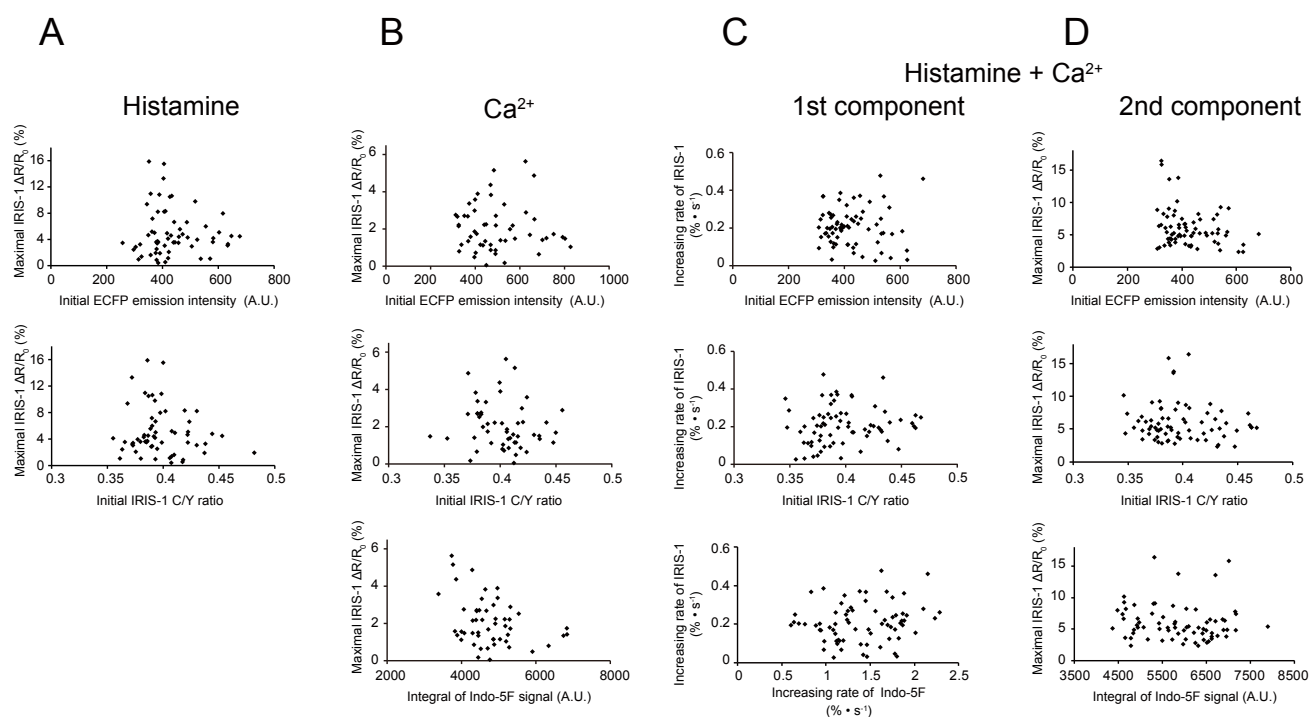

Figure S4

Supplement: Figure S4 — Validation of the IRIS-1 signal changes observed in thapsigargin-treated HeLa cells. (A and B) The maximal IRIS-1 signal changes after addition of 3 µM histamine (A) and 2 mM Ca2+ (B) were plotted against the basal CFP intensity of IRIS-1 (top), resting IRIS-1 C/Y ratio (middle), and integrated value of Indo-5F change for 10 min (bottom). (C) The initial rate of IRIS-1 signal change (1st component) after addition of 3 µM histamine plus 2 mM Ca2+ was plotted against the basal CFP intensity of IRIS-1 (top), resting IRIS-1 C/Y ratio (middle), and initial rate of Indo-5F change (bottom). (D) The maximal IRIS-1 signal changes after addition of 3 µM histamine plus 2 mM Ca2+ (2nd component) were plotted against the basal CFP intensity of IRIS-1 (top), resting IRIS-1 C/Y ratio (middle), and integrated value of Indo-5F change for 10 min (bottom). (PDF) [file pone.0086410.s004.pdf]

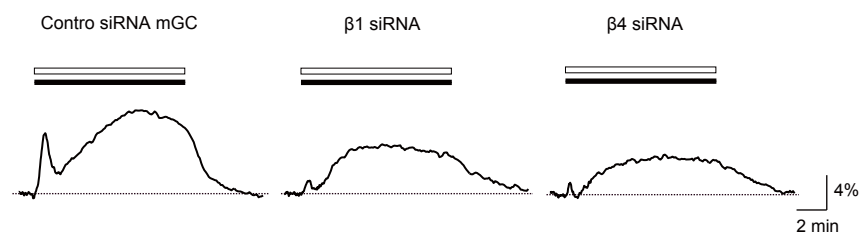

Figure S5

Supplement: Figure S5 — [IP3] changes after histamine stimulation plus [Ca2+] elevation in thapsigargin-treated PLC-β1 and PLC-β4 knockdown cells. Representative traces of IRIS-1 signal changes (ΔR/R0) after addition of 3 µM histamine (horizontal open bars) plus 2 mM Ca2+ (horizontal filled bars) observed in thapsigargin-treated HeLa cells transfected with control siRNA mGC (left), PLCβ1KD siRNAs (middle), or PLCβ4KD siRNAs (bottom). (PDF) [file pone.0086410.s005.pdf]
